# Editorial: Tailoring in implementation science

**DOI:** 10.3389/frhs.2023.1233597

**Published:** 2023-06-14

**Authors:** Bianca Albers, Tim Rapley, Per Nilsen, Lauren Clack

**Affiliations:** ^1^ Institute for Implementation Science in Health Care, Faculty of Medicine, University of Zurich, Zürich, Switzerland; ^2^ Department of Social Work, Education and Community Wellbeing, Northumbria University, Newcastle upon Tyne, United Kingdom; ^3^ NIHR ARC North East-North Cumbria, Newcastle, United Kingdom; ^4^ Division of Community Medicine, Department of Medical and Health Sciences, Linköping University, Linköping, Sweden; ^5^ Department of Infectious Diseases and Hospital Epidemiology, University Hospital Zurich, Zurich, Switzerland

**Keywords:** tailoring, implementation science, implementation strategies, knowledge translation, implementation determinants

**Editorial on the Research Topic** Tailoring in implementation science

It is commonly accepted that a “one size fits all” approach to implementing research-supported interventions (RSIs) in routine health care settings is inadequate. Instead, scholars have called for “tailoring” as a means of improving the fit between an intervention and the context in which it is implemented. Tailored implementation is a prospective process involving the (1) identification and prioritization of barriers and/or facilitators (i.e., determinants) likely to influence the implementation of RSIs and (2) selection, operationalization, and application of implementation strategies likely to address the identified determinants (1). While this promise of tailoring may appear sensible, the evidence of its effectiveness is variable and, at best, modest (2). This hampers our understanding of how tailoring works, for whom, under which conditions, and in what way and of how it can best be applied across the pathways of an RSI to maximize its potential effect. This Research Topic seeks to enhance our understanding of tailoring in implementation science, by drawing attention to the knowledge gaps that surround the concept of tailoring and showcasing real-world examples of practicing tailoring in health care studies.

Out of its eight contributions, three alert to aspects of tailoring that remain insufficiently studied and require further conceptual development. Nilsen et al. start off these contributions by encouraging theory use in tailoring. They argue that successful implementation requires the selection and design of implementation strategies based on appropriate theories to achieve intended individual or organizational behavior changes. The authors emphasize that theory use too limited in scope or derived from inaccurate assumptions about behaviors in question may lead to implementation challenges or failure. They recommend combining insights from various theories to consider different influences on behaviors and to use these insights when developing and tailoring implementation strategies.

Complementary to this behavior focus, Metz et al. remind us that tailoring also requires attending to less visible influences present among implementers and implementing organizations and systems. These are the mental models of and the relationships among implementers and the climate characterizing their implementation efforts, all of which are potential determinants that influence tailoring but are difficult to grasp. The authors therefore call for a “values-driven” approach to implementation through which practitioners’ and communities’ values and guiding principles direct the selection and tailoring of strategies used in implementation.

Two further conceptual contributions focus on the dynamic and complex character of real-world health care settings and highlight the need for tailoring approaches to be able to match these dynamic conditions. In the context of infection prevention and control in hospital settings, von Lengerke et al. emphasize that change typically occurs through a series of courses of actions, unfolding at different parts of an organization, and requiring to tailor to these parts and their actors. Additionally, Haverhals et al. point to the pace at which an organizational context can change, necessitating speed in tailoring, i.e., in understanding and responding to emerging determinants and in adjusting implementation strategies. This therefore calls for the development of more rapid methods for tailoring.

Three empirical study reports illustrate how tailoring can be practiced and expand the range of questions needed to be considered in progressing the tailoring research agenda in the future.

Contributions by Valenta et al. and Potthoff et al. demonstrate the complexity of tailoring when aiming to balance the use of information retrieved through theory, stakeholder involvement and further contextual analysis. Based on the implementation of an integrated care model for stem cell transplantation, Valenta et al. share a comprehensive description of merging insights from a contextual analysis and stakeholder input with considerations about current regulations in adapting an intervention and tailoring supporting implementation strategies. Potthoff et al. share experience with developing a tailored strategy for training general practitioners (GPs) in addressing alcohol use with relevant patients. Their approach combines the use of focus groups to identify GP needs with utilizing the Behavior Change Wheel to determine functions that the training strategy should fulfill. In a self-critical reflection, they point to the missing patient perspective in this tailoring work, and the scarce knowledge that exists about how to tailor to different and conflicting needs of multiple implementation stakeholders. Finally, Leeman et al. based on the scale up of a lifestyle program across health care providers in North Carolina, make the case for aligning tailoring efforts with the stage of implementation. They illustrate how new strategies had to be selected and designed to address determinants specific to a state-wide program scale-up and how previously developed strategies had to be tailored to, e.g., reduce the research team involvement that characterized earlier implementation stages.

**Figure 1:**
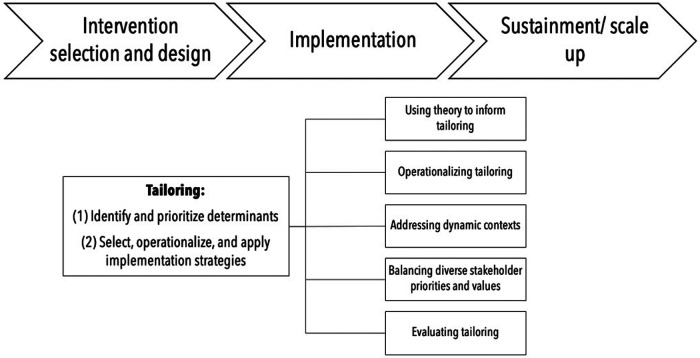
The intricacies of tailoring across implementation stages.

Collectively, these papers reveal central intricacies of conceptualizing, researching, and practicing tailoring in health care (see Figure 1) and the existing chasm between the knowledge base for understanding tailoring and its immediate appeal among implementation researchers and practitioners.

We therefore support the call to action forwarded by McHugh et al. in their contribution to this Research Topic. To strengthen the knowledge base on tailoring, they demand better future reporting of tailoring in combination with expanded research activity in this field and urge researchers to address four questions about tailoring through their work: (1) What constitutes tailoring, and when does it begin/end?; (2) How is tailoring expected to work?; (3) Who and what does the tailoring process involve?; and (4) How should tailoring be evaluated? Based on the contributions to this Research Topic, we suggest adding two further questions to this catalogue: (5) How can the use of theory enhance our understanding of tailoring?; and (6) How can diverging and conflicting stakeholder preferences be managed as part of tailoring? This research agenda is put forward at a critical time in the development of implementation science.

While the field continues to grow, it has developed to a point that merits critical reflection and attention to developing and employing sound scientific methods—of which tailoring is a central element. We hope this Research Topic will inspire researchers to rigorously engage with the topic of tailoring in future activities.
